# Residency to Reality

**DOI:** 10.1055/s-0045-1814456

**Published:** 2025-12-31

**Authors:** Samreen Jaffar, Maneesh Singhal

**Affiliations:** 1 Department of Plastic and Reconstructive Surgery, Aster MIMS, Kasaragod, India; 2 Department of Plastic Surgery, All India Institute of Medical Sciences, New Delhi, India

Completing residency marks a transition from structured training to independent professional practice. Despite demanding schedules, intense clinical work, and academic expectations, residency provides supervision, direction, and structure. It offers a protected environment in which students develop clinical judgment, procedural skills, and discipline. It shapes the professional identity, and moving beyond it introduces new responsibilities. Independent practice requires self-directed decision-making, planning, and accountability, often without the predefined structure of residency.

We have often reflected on how the Indian plastic surgery training program equips residents for the transition to independent practice, its strengths, and areas for improvement.

## Plastic Surgery Training in India

The field of plastic surgery has evolved rapidly over the last few decades. What began primarily as a reconstructive specialty, has now grown into a remarkably diverse discipline adopting considerable technological advancement, be it microsurgery, supermicrosurgery, virtual surgical planning, regenerative medicine, or three-dimensional solutions. This diversity is something to be proud of, but it also challenges our training programs to keep pace.

Over the years, the number and quality of training centers have grown. From a handful of superspeciality institutes a couple of decades ago to numerous MCh and DrNB programs across the country, the expansion has been remarkable.

The enormous caseload in most teaching institutes provides residents with a wide exposure to common conditions like trauma, diabetic foot, head and neck reconstruction, burns, pressure sores, and congenital anomalies. This also gives the confidence to deal with complications, hence equipping residents to be independent early in their careers. Indian trainees are also used to being adaptable and working efficiently, even with resource limitations. The teachers in most institutes are deeply dedicated, and many are pioneers in their fields, allowing students to learn from their vast experience. These intangible elements prepare residents, far more for the realities of practice than is realized while in residency.

Despite the overall progress in resident training, exposure to some subspecialities remains uneven and may not fully reflect the expanding breadth of the specialty. The exposure to advanced technology is also not uniform across centers. Some residents graduate well-trained in certain domains of plastic surgery, but with limited exposure to others. Research exposure too varies between centers, depending on faculty engagement, infrastructure, and mentorship. Structured mentorship and longitudinal evaluation systems are limited, making it difficult for residents to identify areas requiring development early. Areas such as communication, financial literacy, private practice management, medico-legal awareness, and well-being receive relatively little formal emphasis during training.

Many young plastic surgeons step out of their residency, into a world that is very different from their training institute. Along with the inherent responsibility of providing patients with high quality care and having the skill enough to do that well, they realize that there are new challenges to surmount and new frontiers to conquer.

## Residents' Forum: APSICON 2025

Against this backdrop, the APSICON 2025 Residents' Forum was conceptualized with a singular goal: to address the anxieties of young plastic surgeons with practical guidance, wisdom, and experience from some of the finest plastic surgeons in the country. This session was meant to help bridge the gap between *Residency and the Reality* .

The hall was packed with enthusiastic residents and some of the top faculty in the nation. A set of much-debated, often blurry, and confusing topics were taken up for discussion.

Many residents who voiced their concerns shared that, despite rigorous training, they occasionally felt less confident when facing certain subspeciality-specific situations.

Financial considerations were also mentioned. “Given the length and intensity of our training, aligning remuneration with skill acquisition remains a challenge in the early phase of practice,” one participant observed. In addition, questions arose regarding fellowship choices, burnout, and well-being, managing early-career setbacks, and building confidence when stepping into independent practice after residency.

The faculty addressed each of the following topics:

1. Subspecialization: Jack of all trades or master of one?
2. Crossing borders: Fellowships and migration
3. Tackling the corporate world and private practice
4. Pursuing an academic career
5. Opportunities in tier 2 and tier 3 cities
6. Burnout, mental health, and work-life balance

Finally, everything was tied together by a crisp take-home message

The key 10 takeaways from the session were:

1. A strong base of general plastic surgery must be built during residency. Additionally, one must strive to develop their USP, based on individual interest and expected place of practice. *Be a good general plastic surgeon who is a specialist in a particular aspect of it* .
2. Train well to have exceptional personal competence. *In the first five years of your career, choose to work with someone who is significantly more competent than yourself* . Pick a niche and get additionally trained for it.
3. Though the competition is stiff, both from within and outside the community, it is ultimately core competency that will shine through. Competition encourages professional growth when approached constructively.
4. There is nothing wrong with wanting to earn well. *But be patient and keep your needs low, especially early in your career* . It takes 3 to 5 years for your practice to grow and the earnings to match your expectations.
5. There is no substitute to hard work. It is not only important to have the ability to do a particular case well, but to be available to do it, given any hour.
6. Learn to adapt to circumstances, whether in a corporate hospital or in a teaching institute, without compromising ethics or speaking ill of your peers.
7. Seek mentors and collaborations. Form a team of like-minded people.
8. Network and be seen. Speaking about your work not only to colleagues from your own specialty, but also of other specialities is very useful. Spreading awareness about our specialty to the general public and to other specialists is also our responsibility and will go a long way.
9. An academic career is more in the person rather than in the place. Keep up with advances in the field, and work to add to the scientific pool by publishing and teaching peers and juniors.
10. Family time is important. It is also important to have many friends (even outside our fraternity) and to read widely, even outside just academic books.

The 1.5-hour session was special. The energy was infectious with seasoned teachers eager to guide, and young surgeons eager to learn. The Residents' Forum did not end all doubts, but it definitely showed us that the residents' fears are relevant and shared. It also reassured them that they have mentors they can approach when faced with difficult decisions.

## Where Do We Go from Here?

One thing is absolutely certain: *Only when each trainee in plastic surgery does well, our specialty will thrive and grow even beyond what it has in the past* . Strengthening our training ecosystem is critical for long-term advancement of the specialty.

While seniors in our field are keen to help, and juniors are equally eager to learn, a gap still exists. Bridging this requires deliberate, thoughtful intervention.

1. Standardizing training across centers
  No two programs can be identical, but all residents deserve a baseline exposure to every major domain in plastic surgery. Externships and visits to centers of excellence during residency have been promoted by the Association of Plastic Surgeons of India (APSI) to gain exposure in aesthetics, onco-reconstruction, hand surgery, and other niche fields, and most centers are more than welcoming to trainees. The importance of this has been regularly spoken about in APSICON for over a decade now. Yet, at the ground level, there is some resistance to fully adopting this policy in many institutes. Greater institutional support for such opportunities would contribute to a more well-rounded training.
  Encouragement by training institutes to attend national and international meetings and training workshops, early in one's career will definitely go a long way in broadening horizons and building connections. This opportunity too is variable among different centers.
  The APSI can support standardization by encouraging consistent exposure and reviewing training outcomes across centers. An overseeing eye by a formal “Residency Committee” under the APSI can be specifically tasked with auditing and addressing the issues of plastic surgery training in the country.
2. Building a national mentorship network
  Residents should not have to rely on chance encounters for career guidance. A structured mentor-mentee system pairing trainees with senior surgeons from other institutes can fill gaps local training cannot. Such relationships can help young plastic surgeons navigate many professional hurdles with clarity and confidence. In this digital age, this is not a difficult task to accomplish. This can also come under the purview of the proposed Residency Committee, of the APSI.
3. Integrating career skills into residency
  Financial awareness, private practice management, medico-legal understanding, digital professionalism, communication, and counseling must become part of the formal curriculum. Programs like the Residents' Forum can be held more often, and should certainly be an annual occurrence at the APSICON, with more dedicated time. This will serve to normalize talking about the academic expectations of a trainee and the “non-academic” aspects of being a successful plastic surgeon.
4. Strengthening research and innovation
  The high patient volume, in India, positions us uniquely for impactful, practice-changing research. Yet, our research output lags behind. Residents need structured research training, access to biostatistical support, and protected research time to nurture innovation.
5. Cultivating a culture of well-being
  Normalizing conversations around burnout, mental health, and work-life balance is essential. A healthy surgeon is, undoubtedly, a better surgeon.

## Conclusion

Indian plastic surgery has a rich legacy, and some of the most passionate teachers one could hope for. It also has a generation of young surgeons who are driven, thoughtful, and unafraid to innovate. We all recognize that the transition from residency to independent practice is a formative and defining stage in a surgeon's career. Guidance from seniors who have walked this path before us is invaluable.

The APSICON 2025 Residents' Forum was a small but meaningful step toward creating a space where the concerns of trainees and young plastic surgeons could be heard, acknowledged, and addressed. As a freshly graduated plastic surgeon, I left that session with renewed direction and optimism, and I am sure many others did too.

The problems and challenges are real, but there is no use of spreading negativity about them. We must work hard, hone our skills, and effectively collaborate, to rise to the challenge. The future of our specialty will not be shaped solely by advances in technique or technology but by how effectively the next generation of plastic surgeons is trained, supported, and empowered. It is our collective responsibility not just to excel individually but to build a strong, supportive ecosystem for the generations to come.

## Credits

The successful execution of the Residents' Forum was made possible through the collective guidance, support, and vision of the faculty. We extend our sincere appreciation to Dr. Dushyant Jaiswal and Dr. Veena Singh for planning and steering the program, and to the distinguished speakers and panel members: Dr. Surajit Bhattacharya, Dr. Raja Shanmuga Krishnan, Dr. G. Karthikeyan, Dr. Sudhir Singh, Dr. Prabha Yadav, Dr. S. Raja Sabapathy, Dr. V. Bhattacharya, Dr. Karoon Agrawal, Dr. Brijesh Mishra, Dr. Sheeja Rajan, and Dr. Mukund Thatte, for their invaluable contributions, thoughtful discussions, and their commitment to guiding the next generation of plastic surgeons.

